# Pressure Anesthesia

**Published:** 1906-02-15

**Authors:** Geo. Zederbaum


### PRESSURE ANESTHESIA.

BY GEO. ZEDERBAUM.

Read before the Detroit Dental Society, November 9, 1905.

The subject of pressure anesthesia is as yet in its infancy, but it is of very great importance to the profession eve- at this, its experimental stage. To properly prepare a deep seated cavity in a sensitive tooth, to excise a crown or to remove a nerve, all painlessly and without the risk of after effect, is what we are after, and, in my opinion, our goal is not far distant.

What is pressure anesthesia? I would define it as the act of introduction, by mechanical means capable of exerting an enormous pressure, of a fluid medicament into the tooth structure, the medicament finding its way into the dentinal tubules and thence into the pulp proper; thus, rendering the whole organ insensible to all pain for a fixed period. Up to the time of the introduction to the profession of high pressure syringes, many of us knew of pressure anesthesia in its primeval stage, *i. e.*, a piece of spunk saturated with a cocain solution was carried to the exposure, this was covered with a piece of vulcanizable rubber and pressure was then exerted over all for a brief period, when, as a rule, all precautions being taken, the pulp could be painlessly withdrawn from its bony environment, This method was used by us for but one purpose, that is. for the painless removal of pulps only after their exposures had previously been made, either naturally by decay, or artificially by burs. This worked admirably in most anterior and some posterior teeth when the operator succeeded in confining the medicament to the pulp chamber. The pulp was removed painlessly and many filled the canal and the tooth at the same sitting which procedure, by the way, I never approved of for reasons seen later. This was, of course, all right so far as it went, but sitting and enduring tortures while exposure was being made is more than any one of us would have been willing to undergo. So we soon heard of high pressure syringes, which would, to a great extent, do away with the pain incidental to drilling through the dentine. I am not here to advertise any one of these instruments, as the one that may prove successful in my hands, may not in yours; but the main object of all of these is to be able to exercise sufficient steady pressure to force the medicament into the tooth structure. As you all are no doubt more or less familiar with them, I will not go into minute description suffice to say that the Beekton-Dickinson works with direct pressure. Dr. Higgins on a thumb screw and spring principle; the Wilcox-Jewett has a ratchet force; the Meyers a piston leverage; the Sapp screw principle; and the United States, works by a plunger and direct pressure. I have used most of these and, while they all have good points, none of them will do everything we might expect them to do, judging from their advertising matter. Many present would be willing to testify that they do not always work to perfection and that there is still much lacking; but after all is it not our failures that teach us? Is it not our failures that make us study why they were such, and is it not through this study we incidentally learn the truth and under what conditions we may reasonably expect to meet with success?

All the instruments mentioned are made on the same principle and require the same preliminary steps. I will enumerate these in their respective order. Clean teeth within the area of operation: Apply rubber dam and make a pit, in a most convenient place for direct pressure, drill through the enamel and just into the dentine with a sharp and correct size bur; forcibly blow out all the chips, and apply syringe point, making as nearly as is practicable a water-tight joint. The medicament must be securely confined. Hold the instrument steady and exercise a steady pressure from two to two and a half minutes, which is all that is usually necessary and you can then proceed with excavating the cavity; and in the majority of cases, there will be no sense of pain. For the removal of the pulp I would repeat the operation, as in many cases, this second application proves to be necessary. This is all there is to the modus operandi; varied, of course, to suit each individual case; always keeping in mind that we must have surrounding walls that will not permit leakage and that when these are absent we must supply them, or direct our efforts to some other portion of the tooth.

Now, then, we come to the question of what is the best to use for the obtunding solution. Most of the high pressure syringe patentees sell obtundent tablets, the formulas for which are known to themselves alone; but I am quite satisfied that they all contain more or less cocain for their active principle. I have tried a 3 percent Beta Eucain solution (Shering) with some degree of success; also a 3 percent solution of cocain in 33⅓ percent solution of formaldehyde in alcohol; also almost a saturated solution of cocain in adrenalin chlorid and a 4 percent solution of cocain in chloretone. I have also used nothing but distilled water, which proved most clearly to my mind that the distilled water (or rather the pressure from it) will cause anesthesia; but as Dr. Loeffler (*Dental Digest*, April 1905) says: "Distilled water used under pressure will produce painful anesthesia, that is, after the effect of anesthesia wears off there will be a reaction in direct proportion to the intensity of anesthesia produced, manifesting itself in dilation of the blood vessels and considerable accompanying pain." In my hand 2 and 4 percent solution of cocain hydrochlorate in chloretone has proved very satisfactory. Chloretone, as we know, is a derivative of chloroform and acetone, and has antiseptic, anesthetic and hypnotic properties. Parke, Davis & Co. put up 2 and 4 percent solution of cocain in 84 percent chloretone. This concern says: "The presence of chloretone augments the anesthetic effect, and prevents decomposition and deterioration" (both very desirable qualities).

Now let us see what becomes of the cocain solution when same is used under pressure as described. We do not know the exact physiological reaction which takes place, but we are told and strongly believe that cocain is a poison of protoplasm, and has a selective affinity for the contraction of blood vessels, and it is by these two actions that we account for the local anesthesia produced. I, for one, am satisfied that any liquid forced into the tooth structure finds its way through the protoplasmic contents of the dentinal tubules into the pulp and odontoblastic nerve filaments, and also into the pulp proper, engorging the vascular tracts until either extravasation, systemic absorption or bodily removal takes place.

I have been making some microscopical tests to demonstrate the probable course of cocain, which experiments may prove of interest to you. First, an examination of minute cocain crystals was made to familiarize myself with their shape, translucency, etc. Next I evaporated on a slide one drop of almost saturated solution of cocain in formaldehyde and alcohol and noticed the residue with naked eye first; and then, using two-thirds objective, which revealed the beautiful cocain crystallization with its characteristic prismatic radiation. Scattered here and there throughout the field minute undissolved particles were easily discerned. Next I removed an apparently healthy live pulp from an upper central without the use of local anesthetics (patient being under the nitrous oxide). The pulp was handled with clean (I would not say sterilized) broaches and was placed under the microscope. Small amount of blood was visible. I teased this nerve tissue and then dessicated the same, and in neither condition could detect any particles resembling cocain crystals. Microscopical examination of a pulp removed after arsenical application, although entirely modified structurally, did not show these crystals. I then conducted a long series of examinations of different specimens of pulps that had been removed under pressure anesthesia, using an almost saturated solution of cocain in 33 percent solution of formaldehyde in alcohol, this menstrum being used in preference for its high penetrating quality, and, in each instance, I found along the blood tracts minute granular deposits, which I believe I can reasonably call cocain. Further, upon teasing and macerating a number of these pulps en-masse, the moisture obtained was collected and dried, and upon microscopic examination, prismatic films radiating in all directions and comparing favorably with the slide of evaporated cocain examined previously, was noticed. So much for the course of cocain in pressure anesthesia. My opportunities for research, regretfully, are very small, being located away from a great institution of learning, and where I cannot do more along this line from lack of necessary apparatus. Dr. Ottolengui suggests the use of polariscope; and no doubt, gentlemen, some of you at the Univeısity ought to, and soon probably will, take care of this interesting question.

Somewhere in the beginning of this paper I said that there are many conditions where we can not make pressure anesthesia effective. I will now briefly enumerate some: Teeth of old persons; teeth of inveterate tobacco chewers; worn, abraded and eroded teeth; teeth with extensive secondary calcific deposits; teeth whose pulp canals are obstructed by pulp nodules; teeth with metallic oxides in tubules; teeth with leaky old fillings and badly calcified teeth. All of these conditions have, in the main, one and the same cause of failuıe; namely, clogged tubuli, whether from abrasion or erosion, where nature seals these tubules to do away with sensitiveness and prolonged usefulness of the organ; or whether from secondary dentine deposit, due to some trumatic injury; or whether from old fillings. In most all such cases no amount of persistent pressure will prove successful.

On the other hand children's teeth, where dam can be applied; teeth of young peısons; properly calcified teeth; teeth that have never been filled, readily respond to pressure anesthesia.

Now, then, comes another important question. Do we endanger the vitality of otheı wise healthy pulps when we use "cocainized" (if you'll permit the term) pressure anesthesia simply for the purpose of painless tooth excavation? The recuperative property of the nerve tissue due to its abundant vascularity helps to minimize the dangers in a measure, but, gentlemen, should we not know the exact strength of the solution of such a drug as cocain? Should we not know how much of any strength we could use with impunity, and when we had exercised enough pressure to bring about the desired effect without forcing the drug into the perapical space? Only by the exercise of such care can we avoid pulpitis and a train of complications beyond the foramen. When we bodily remove a pulp, and the usual hemorrhage ensues, we eliminate a good many sequelae by allowing the engorged vessels to empty and to rid themselves of noxious substances that may have collected there. In simple desensitising for excavation, however, we have no similar advantage.

What, then, of possible infection or after effects of a troublesome character? Cases are on record where trouble ensued and where no amount of counter-irritation and palliative treatment relieved the condition and ultimate death of the pulp resulted.

This question I fear will be left without an answer for some time to come but it may serve as a stimulous to still further thought on this important far reaching subject of pressure anesthesia.

Gentlemen, I am sure you are getting tired of this somewhat exhaustive treatise, but we are drawing to an end and I want a sharp discussion on every point. Let us bring out all there is and not until we do will I be content with my limited effort.

Before closing I wish to say a few words regarding the in-advisability of filling canals immediately, or at the same sitting after the pulp extirpation. I have examined many pulps as they came out on the broach and I am satisfied that, part of the time, we do not remove the whole of the pulp from the root even though we are confident. It is the custom of many to immediately fill the canals after the hemorrhage has apparently subsided. Did you ever have your patient ask you why this canal filling process hurt so, when the nerve was out? Did you ever have him return some two or three days after this wonderful operation and complain of soreness and uneasiness, and after all else failed, you opened up again, reluctantly but wisely? I think the canals should be left unfilled for a few days at least. Clean them out as thoroughly as possible, and apply some dry dressing and await further developments. Then, after a lapse of a few days, there being no secondary hemorrhage and no perceptible soreness on percussion, you may proceed with a fair degree of assurance of no further troubles, such as hemorrhages or other hyperaemic reactions or infections.

Finally, I wish to state that there are many among us who disregard all precautions and are so enthusiastic over pressure anesthesia, that they ignore the fact that this method is practically as yet in its experimental stage. While we meet with apparent success in a fair percentage of cases, this method is not without its dangers and there are not a few cases in which failures in one form or another resulted, many of which we may not see.

### DISCUSSION

Dr. Loeffler: The first point I should make in regard to pressure anesthesia perhaps is that the principle of hydraulics is not always definitely understood. In a recent journal I read an article by a man that ought to know about these things, and he illustrated the use of an ordinary dental syringe, explaining the technic by carefully applying the point to a small opening and then, using the force of an ordinary man, you can readily see what an enormous pressure you get at the little end or point of the syringe. Any syringe is simply the principle of hydraulics reversed; we get comparatively little pressure in comparison to the surface. On this account we have developed the compound syringe for high pressure anesthesia. We cannot increase the pressure unless we make the barrel smaller or unless we increase our leverage, and that is what we do in the screw or lever principle. In the bicycle pump, the larger the barrel the more force you must use, and the smaller the barrel used the more efficiency from the pressure. A few years ago Dr. Meyers, of Cleveland, discovered this method for taking out pulps without pain by injecting cocain under high pressure. It is not necessary at all times to have an exposure, as we can now force the cocain right through the dentine to the pulp. If we can get the proper pressure, after making a cylindrical opening into the dentine, and fitting the opening carefully with vulcanized rubber, exert a great pressure on the rubber and force the cocain into the pulp as well as with the syringe, and that too without a pulp exposure or very near approach. I have in that way anesthetized the pulp without making an exposure or giving unnecessary pain excepting at the beginning. We must confine our liquid and our pressure in order to do it. In our clinic we have a Meyers, a Wilcox and the Sapp syringe and another, the Dohme, I think, which has a special attachment for hanging on the tooth. We have there a good many failures as well as successes in removing pulps, because we get teeth that have been irritated for a long time, secondary dentine, and pulps to take out for old people where the cocain will not work. Why does it not work? That is the reason Dr, Zederbaum and others are doing some work on that line. to see what actually becomes of the cocain and if it actually gets into the pulp or what becomes of it. I have, thus far, been unable to tell just what becomes of it. We can not see it penetrate into the dentine, yet we actually know that the cocain does get into the pulp through a considerable thickness of dentine. I have tried alcoholic solutions and chloroform, because you know the molecules of alcohol are smaller than those of water and it penetrates better. I learned that we must use a good, clean solution; anything that hampers its entrance into the tissue will hinder its efficiency.

Another important point is that the point of application must be so the point will fit, a surface that is covered by enamel so as to hug it as tight as possible. The dentine will sometimes give way, so that there are many things to contend with. At the point of application we may be handicapped in getting a good location for the pit. I remember when cataphoresis was first used to force cocain into the dentines, that the question was asked: "How will it affect the pulp?" Won't the pulp die afterwards? Dr. Nancrede, when I told him about that, said: What will be the after effects? Why do men ask these questions? Simply because they naturally expect an after effect; we do from any operation. With the teeth this is a serious consideration, for we can take out a pulp in just a few minutes blanched white and suddenly you have quite a profuse hemorrhage. I most heartily agree with Dr. Zederbaum that we must give vent to this primary hemorrhage, the reaction. Unless we take care of that we are apt to have trouble. Unless it is taken care of by the tissues or lymphatics, we are apt to have trouble about the absorption of this blood. The tooth ought to be left for a day or two after with some application to take care of this hemorrhage.

To get the most good out of this subject, I think we ought to all help along this line. We all want to know about these things, and it is not fair for anyone or any few to spend their time trying to find out, not alone for their own glory, because often they get very little out of it.

Dr. Hampton: I would like to ask the question why it is that the pressure anesthesia is not effective in persons who are inveterate tobacco chewers, also in old persons. Probably in the case of old persons because the pulp canals have become small, due to the deposits on the inside, and therefore we have a greater distance to go, and perhaps the tooth is not as vital as it is in youth or middle age, but I do not quite see why it is that inveterate tobacco chewers should make us greater trouble than others. In the case of bicuspids I find that in using high pressure anesthesia when we have a proximal cavity of good size, it is better to make the entrance at the side, and not directly in the center. In the embryonic stage we have two centers of calcification of the bicuspid tooth, and as they fill in around these centers they grow toward each other. Toward the center of the proximal surface we have tubuli which are not as straight and the pulp is thinner at the center of the proximal surface than toward the lingual or buccal surface. Perhaps that is why we get better effects than by going directly toward the center of the tooth. So many times we have a leathery decay in the cavity and it is exceedingly painful to remove, it is so sensitive, so I sometimes use my pressure syringe right into that leathery decay just removing what will come out readily, and then inject directly through it.

During the last two or three days I telephoned to a number of dentists in the city to find out what they have experienced in regard to their after effects, and have not found, so far, any after effects that were especially detrimental. One dentist said: I expect in a year or two to harvest a crop of troubles with other dentist's and my own work, I expect to have a number of dead pulps. One of the dentists said he liked to use a little adrenalin also with his solution when he used the pressure anesthesia, especially if he has an actual pulp exposure to prevent the hemorrhage. If you can take out the pulp thoroughly bleached and have no hemorrhage, you do not need afterwards to force a broach into the very fine root canal.

Dr. Thompson: As for me and my house, unless a pulp removal is indicated in a perfectly healthy tooth for crown, bridge work or something of that kind, there has been no trouble from inflammatory processes of that kind and I do not want any pressure anesthesia in mine. Some of the most disastrous and unpleasant results that I have ever had in my experience with this subject have been directly as a result of pressure anesthesia used on the exposed pulp and the subsequent excision of same. I had a tooth with pulp exposed in my own mouth that was aching fearfully, and I drifted into the office of a friend to stop it. He deliberately took a hypodermic syringe and solution of cocain and injected each root canal and I thought I should die;I lost the tooth. I never could get that tooth so that it would remain sealed up for more than four weeks at a time. It finally broke off, leaving the roots there loose and I had to have them out. I

am heartily in favor of pressure anesthesia or application of cocain in a perfectly healthy pulp. If the pulp comes out quite clean and there is no hemorrhage you do not need to worry so much about the results, but I believe it is best to let it remain unfilled for a day or two with an antiseptic dressing. A great many times the pulp will be severed inside the canal or shreds will be left adhering to the walls of the canal, and cause inflammation either of greater or less importance.

Dr. Buttrick: Dr. Hampton called me up by telephone and inquired if I had any disastrous effects after pressure anesthesia. I told him that I did not use the pressure syringe except where I expected to extirpate the pulp. I think it must have a profound reaction and I shall be surprised if we do not have to treat some alveolar abscesses in a year or two. It seems to me that by shutting off the blood supply for even a short time you must threaten the life of the pulps, or will there be a return of the circulation again? Dr. Hampton says he used it for obtunding dentine. I do not believe in injecting into anything except sterilized dentines, because if you drive the bacteria into the pulp you are certainly going to have trouble. Get the rubber dam on and by plenty of carbolic acid and hot water disinfect the dentine and then use your cocain if you want.

Dr. Zederbaum: Regarding tobacco chewers it probably is the nicotine or smoke debris present that is the obstacle to the pressure method. The direction of the tubuli in the bicuspid may influence the penetration, inasmuch as the two lobes converge at that point, and the injection at an angle would be advisable. It is not reasonable to expect that in forcing the solution into the tubuli we may force out the soluble protoplasmic contents of the tubuli into the pulp, or because of the contact with such medication as we use, either the simple cocain solution or combined with adrenalin and other medicines, we may change the protoplasmic fluids to such an extent as to get some coagulation or disintegration, and it is not at all unlikely that what I have seen under the miscrocope may have been such.

To have success with pressure anesthesia I agree with Dr. Thompson. The pulp tissue should be healthy when injected preferably. There is little question about the fact that the pulp tissue contents and the dental tubules are continuous, because we know after the pulp is dead we can drill into the dentine anywhere without a particle of pain, when we have a cavity to prepare. Many times when I have a sensitive cavity which I cannot excavate by usual methods, it is too sensitive to work with the warm air and cocain, I resort to the pressure anesthesia, in these cases I make it a rule to make the injection in an inconspicuous place, as well as where the most direct pressure can be obtained in the least sensitive part of the cavity if practicable.

One of the greatest difficulties with these syringes is to hold them steady long enough to secure complete results. In some cases you cannot hold the instrument two or three minutes and exercise the pressure necessary.

The quantity of cocain to be used is a very hard matter to determine. I see Shieffelin & Co., of New York, are manufacturing a cocain tablet which they call eurocain, in the shape of little pellets. This is a veiy good idea because you can easily secure just the amount you want to use; it should be very handy if the quality of the cocain is good.

Regarding complete nerve removal and tooth saving at one sitting, it may be all right for that class of patients which never come back. Some, you know, come in and we relieve their tooth ache and they never come back again.

Sometimes it is advisable to remove the pulps from deciduous teeth and we have considerable trouble. Pressuie anesthesia works very well here, generally it is unnecessary to use the syringe in these cases. I should not advise filling the canals of such teeth immediately.

I do not know whether many of you have tried to remove pulps without local anesthetics, but under the gas. It is a difficult task to get many of the patients to submit themselves to take gas, but if you can get them to do that, you can extract your nerve without any tiouble at all in a very short time. It is not necessary to put the patient entirely under the influence of the gas. I have taken out five or six at a time without the patient knowing anything about it.

In Germany recently a dentist advocated a new method, using a solution he called eusaning. He used it in the same manner as you would local anesthetic before extracting the tooth; apply needle point in the direction of the apex. We must know the direction of the root in the first place, and we are apt to have the same trouble as we have in the anesthesia method. Cataphoric infiltration of the tissues, by means of electricity, is a good and safe method, but the disadvantage of this method is that the majority of us have not sufficient knowledge of electricity to satisfactorily use it.

